# Adenosine and the Auditory System

**DOI:** 10.2174/157015909789152155

**Published:** 2009-09

**Authors:** Srdjan M Vlajkovic, Gary D Housley, Peter R Thorne

**Affiliations:** 1 Department of Physiology, Faculty of Medical and Health Sciences, The University of Auckland, New Zealand; 2 Discipline of Audiology, Faculty of Medical and Health Sciences, The University of Auckland, New Zealand; 3 Department of Physiology, University of New South Wales, Sydney, Australia

**Keywords:** Adenosine, adenosine receptors, cochlea, hearing, deafness, oxidative stress, noise, ototoxicity.

## Abstract

Adenosine is a signalling molecule that modulates cellular activity in the central nervous system and peripheral organs *via *four G protein-coupled receptors designated A_1_, A_2A_, A_2B_, and A_3_. This review surveys the literature on the role of adenosine in auditory function, particularly cochlear function and its protection from oxidative stress. The specific tissue distribution of adenosine receptors in the mammalian cochlea implicates adenosine signalling in sensory transduction and auditory neurotransmission although functional studies have demonstrated that adenosine stimulates cochlear blood flow, but does not alter the resting and sound-evoked auditory potentials. An interest in a potential otoprotective role for adenosine has recently evolved, fuelled by the capacity of A_1_ adenosine receptors to prevent cochlear injury caused by acoustic trauma and ototoxic drugs. The balance between A_1_ and A_2A_ receptors is conceived as critical for cochlear response to oxidative stress, which is an underlying mechanism of the most common inner ear pathologies (e.g. noise-induced and age-related hearing loss, drug ototoxicity). Enzymes involved in adenosine metabolism, adenosine kinase and adenosine deaminase, are also emerging as attractive targets for controlling oxidative stress in the cochlea. Other possible targets include ectonucleotidases that generate adenosine from extracellular ATP, and nucleoside transporters, which regulate adenosine concentrations on both sides of the plasma membrane. Developments of selective adenosine receptor agonists and antagonists that can cross the blood-cochlea barrier are bolstering efforts to develop therapeutic interventions aimed at ameliorating cochlear injury. Manipulations of the adenosine signalling system thus hold significant promise in the therapeutic management of oxidative stress in the cochlea.

## INTRODUCTION

The cochlea of the auditory system is one of the masterpieces of the human body. Its unique structure enables analysis of complex sounds by translating them into bio-electrical messages delivered to the brain. Sound energy is collected and focused by the outer ear, and directed through the middle ear to the cochlea. Here the mechano-electrical transduction processes take place whereby sensory hair cells detect each frequency component of the stimulus and convert these into neural impulses which are transmitted to the brain. The complex auditory pathways from the brainstem through to the auditory cortex enable analysis of the sound to provide information on aspects of the sound stimulus such as the localisation of sound sources, frequency discrimination, and detection of complex patterns characteristic of speech.

Hearing is a key sense in human communication and the loss of hearing can be very socially debilitating. According to the World Health Organization (WHO), hearing loss is the 6^th^ ranked cause of the global disease burden significantly affecting over 250 million people worldwide. Hearing loss from noise exposure (NIHL) is a leading occupational disease, with up to 5% of the population at risk. About 80% of the cases of hearing loss occur in people over the age of 50 years and it is set to become a major disability as the population ages with estimates of 26% of the population affected by 2050 (Access Economics, 2006). Hearing loss may have a major impact on the quality of life and psychological well-being. Communication difficulties and poor psychosocial functioning may lead to depression, anxiety and possibly cognitive decline [35]. Even though acquired hearing loss is often preventable, finding safe and effective treatments for cochlear injury and subsequent hearing loss is a holy grail of auditory research.

## STRUCTURE OF THE INNER EAR

**Fig. (1):**
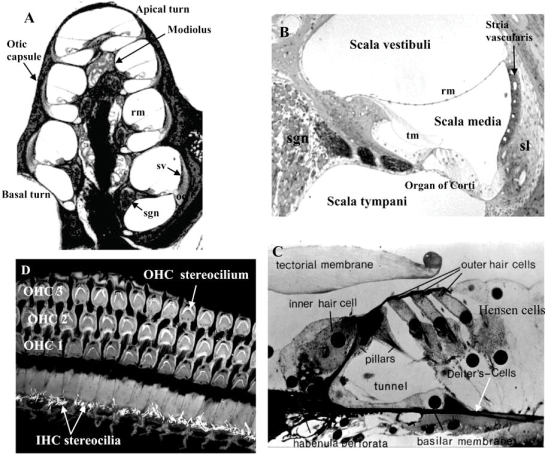
Mammalian cochlea. **A**) A cross section through the cochlea shows the central bony part (modiolus) which contains blood vessels and the auditory nerve, and the cochlear coiling divided into three compartments: scala vestibuli, scala media and scala tympani. Scala vestibuli and scala tympani contain perilymph. The organ of Corti (oc) sits on the basilar membrane and bathes in the potassium-rich fluid of the scala media (endolymph). **B**) Sensory tissues in the cochlea are located in the organ of Corti, secretory tissues is represented by the stria vascularis (sv) and primary auditory neurons are located in the spiral ganglion. High potassium content of the endolymph is maintained by secretion from the stria vascularis supported by the spiral ligament (sl). Afferent innervation of the inner and outer hair cells is provided by the spiral ganglion neurons (sgn). **C**) The organ of Corti comprises sensory cells (inner and outer hair cells) and a variety of supporting cells (Deiters’, Hensen’s and pillar cells). **D**) Surface preparation of the organ of Corti stained with phalloidin showing three rows of outer hair cells and one row of inner hair cells with the apically located stereocilia responsible for mechano-eletrical transduction. Abbreviations: IHC, inner hair cells; OHC, outer hair cells; rm, Reissner’s membrane; tm, tectorial membrane.

The ear is divided into three main compartments, the outer, the middle, and the inner ear, with the latter containing the balance (vestibular) and hearing (cochlea) organs. The vestibular part of the inner ear comprises the sacculus, utriculus and the semi-circular canals, each containing one of the organs of balance. The cochlea is encased by the otic capsule which spirals around the modiolus, a conical bony structure (Fig. **1**). It has three fluid-filled compartments known as the scala vestibuli, scala media (cochlear duct), and scala tympani [22,82]. The scala vestibuli and scala media are separated by Reissner’s membrane, whereas the scala media and the scala tympani are separated by the organ of Corti and basilar membrane. The fluid within the scala vestibuli and scala tympani, known as perilymph, is a typical extracellular fluid, whereas the scala media is filled with potassium-rich endolymph, an intracellular-like fluid. Cochlear fluid homeostasis and generation of the endocochlear potential, a positive electrical potential within the scala media which is the main driving force for sensory transduction, are critical for normal cochlear function [116]. The stria vascularis and spiral ligament, which comprise the lateral wall of the scala media, are actively involved in these processes [82,116].

Resting upon the basilar membrane is the organ of Corti, the sensory organ of hearing (Fig. **1**). The organ of Corti is a highly differentiated sensory epithelium, consisting of specialised hair cells interdigitating with various types of supporting cells [22,82]. Two types of hair cells exist – inner hair cells (IHCs), which are the primary sensory cells, and outer hair cells (OHCs) that enhance the sensitivity and frequency selectivity of hearing through an electromotile process. Occupying the apical surfaces of the hair cells are cellular projections known as stereocilia, and these are interconnected by tip links and side links [22,82]. It is the stereocilia that mediate the transduction of complex sound waves into electrical activity in the auditory nerve. The basilar membrane is a hydromechanical frequency analyser that encodes high frequency sound at the base and low frequency sound at the apex [21]. Movement of the basilar membrane results in bending of stereocilia on hair cells and opening of mechanically-gated transduction ion channels [82]. Influx of potassium ions from endolymph through these transduction channels depolarises the inner hair cells, which in turn activate voltage-gated calcium channels in the basal pole of the cell. Calcium entry triggers the release of glutamate, which generates nerve impulses in the primary auditory neurons located within the spiral ganglion. The inner hair cells thus perform the crucial process of sensory transduction in the cochlea that initiates the activity in the spiral ganglion afferent neurones.

## PURINERGIC REGULATION OF HEARING

Extracellar purines such as adenosine and adenosine 5’-triphosphate (ATP) are intrinsically related molecules that regulate a number of physiological processes in the auditory system, typically acting in a paracrine or autocrine manner. This purinergic signalling system is very sensitive to changes in the extracellular environment (e.g. due to noise, hypoxia, ischemia, trauma) and promptly responds to them. Adenosine and ATP appear to be important signalling molecules in pathological conditions, when the extracellular levels of both are elevated in the cochlea. This review will highlight purinergic regulation of hearing function with an emphasis on the role of extracellular adenosine in the most common inner ear pathologies.

## ATP SIGNALLING IN THE COCHLEA AND HYDROLYTIC CONVERSION TO ADENOSINE 

ATP can be released from cochlear tissues under stressor conditions such as acoustic overstimulation [73], with release sites identified in the organ of Corti [115] and within vesicles in the marginal cells of the stria vascularis [73,117]. Connexin and pannexin hemichannels are likely to be principal conduits for ATP release [123]. ATP released from damaged cells triggers Ca^2+^ mobilization and a regenerative release of ATP from supporting cells [34]. During the early stages of cochlear development supporting cells of the organ of Corti also rhythmically release ATP to initiate spontaneous action potentials in the auditory nerve before the onset of hearing, pointing to their role in the development of central auditory pathways [103]. Released ATP acts on P2 receptors which also respond to other extracellular purines and pyrimidines (ADP, UTP, UDP). ATP-gated ion channels (P2X receptors) and G protein-coupled P2Y receptors are differentially distributed in cochlear tissues with distinctive roles in auditory neurotransmission [45], sensory transduction [46] and the maintenance of cochlear homeostasis [14,58,101].

Endogenous ATP levels in cochlear fluids are tightly controlled by ectonucleotidases [71,107]. Ectonucleotidases are surface located enzymes hydrolysing extracellular nucleotides to their respective nucleosides. Several groups of enzymes are involved in ATP breakdown to adenosine, including ectonucleoside triphosphate diphosphohydrolase (E-NTPDase) family, ectonucleotide pyrophosphatase/phosphodiesterase family (E-NPP) and alkaline phosphatase [87, 124]. Principal hydrolytic enzymes in cochlear fluid spaces are NTPDases [109,110,112]. Surface-located NTPDase1, NTPDase2, NTPDase3 and NTPDase8 have a capacity to hydrolyse ATP to AMP [87]. Dephosphorylation of AMP to adenosine by ecto-5’-nucleotidase (CD73) represents the final step of the ectonucleotidase cascade. Ectonucleotidases thus limit ATP and ADP spatio-temporal activity and convert the P2 receptor into P1 (adenosine) receptor environment.

## ADENOSINE RECEPTOR EXPRESSION AND DISTRIBUTION IN THE AUDITORY SYSTEM

A family of four adenosine receptors (A_1_, A_2A_, A_2B_, A_3_) is expressed in the rat cochlea [113]. These G protein-coupled receptors utilize different intracellular signalling pathways. The classical pathway of adenosine signalling is through inhibition or stimulation of adenylyl cyclase, but other pathways involving intracellular Ca^2+^ release, phospholipase C (PLC), and mitogen-activated protein kinases (MAPK) are also essential [48]. The A_1_ receptor and A_3_ receptor couple to G_i/o_ proteins that inhibit adenylyl cyclase, resulting in increased activity of PLC. A_2_ receptors stimulate adenylyl cylase *via* G_s_ or G_olf_ proteins. Stimulation of the A_2_ receptors leads to formation of inositol phosphates to raise intracellular calcium and activate PKC *via* pertussis toxin-sensitive Gα 15 and Gα 16 [74]. Adenosine A_3_ receptor stimulation directly activates PI3 kinase as demonstrated by dose-dependent phosphorylation of PKB/Akt which is a downstream target of PI3 kinase [48]. Activation of PKB may be an underlying mechanism of anti-apoptotic activity of A_3_ receptors, due to direct phosphorylation and inhibition of the Bcl-2 family member BAD [54].

All four adenosine receptors couple to ERK1/2 MAPK [48], whilst A_2B_ and A_3_ receptors can also activate JNK and p38 [29,39]. MAPK signalling represents an important pathway for G protein-coupled receptors to modify gene transcription of a series of transcription factors [67]. Stress-activated pathways contribute to pathophysiological responses to stress and apoptosis of ROS-damaged cells [38]. In the cochlea, JNK inhibition protects against hair cell death and hearing loss induced by acoustic trauma and aminoglycoside antibiotics [114].

High affinity adenosine receptors (A_1_, A_2A_, A_3_) are differentially localised in cochlear tissues [113]. A_1_R is distributed in the organ of Corti and spiral ganglion neurons. Within the organ of Corti, A_1_R are expressed predominantly in the supporting Deiters’ cells and the inner hair cells (IHC). A_2A_ receptors are localised to the organ of Corti, spiral ganglion neurons, root region of the spiral ligament and the cochlear blood vessels. The A_3_ receptor is predominantly expressed in the inner and outer hair cells and supporting cells of the organ of Corti, including the Deiters’, Hensen’s, Claudius and pillar cells, as well as the epithelial cells lining the endolymphatic fluid space (inner and outer sulcus cells) and interdental cells of the spiral limbus. Cell bodies of the spiral ganglion neurons also exhibit strong A_3_R-specific immunoreactivity [113].

On the basis of immunohistochemistry there is good evidence that the inner hair cells, supporting Deiters’ cells and spiral ganglion neurons are the dominant cells which express multiple adenosine receptors (Table **1**). The localization of adenosine receptors in these cellular regions, which are important for sound transduction, auditory neurotransmission and cochlear micromechanics, implicates adenosine signalling in the modulation of sound detection and hearing sensitivity. The expression of A_1_, A_2A_ and A_3_ receptors by inner hair cells is consistent with adenosine-induced elevation of intracellular Ca^2+ ^in these cells of the guinea-pig [23]. A_1_ and A_3_ receptors have also been suggested to have an important role in presynaptic regulation of glutamate release from the inner hair cells, consistent with their role in modulating glutamate release in brain neurones [17,18].

The cell bodies of the spiral ganglion neurones express A_1_, A_2A_ and A_3_ receptors which may fulfil different roles according to the level of activation. The main role of adenosine in other tissues is to inhibit neuronal excitability and synaptic transmission, acting predominantly on A_1_ receptors [24]. A_3_ receptors may also have a role in synaptic transmission similar to A_1_ receptors [10,16], whilst A_2A_ receptors facilitate the release of excitatory neurotransmitters [17]. However, although these adenosine receptors are expressed in spiral ganglion neurones their functional role in these cells is still to be determined.

Adenosine receptors have also been identified in parts of the central auditory nervous system. A_1_ adenosine receptors are expressed in the dorsal cochlear nucleus, superior olivary complex, inferior colliculus, and auditory cortex in the temporal lobe [86]. In most of these regions, A_1_ receptors are located on cell bodies and axons, supporting the concept that A_1_ receptors act both pre- and post-synaptically. A_1_ receptors are heavily distributed in regions rich in excitatory amino acids, implying that A_1_ receptor stimulation modulates excitatory neurotransmission. In contrast to the A_1_ receptors, the distribution of A_2A_ receptors in the central nervous system appears to be more discretely localised to parts of the inferior colliculus and layer VI of the auditory cortex [88].

## ADENOSINE METABOLISM IN THE COCHLEA

**Fig. (2). Adenosine production, transport and metabolism.**
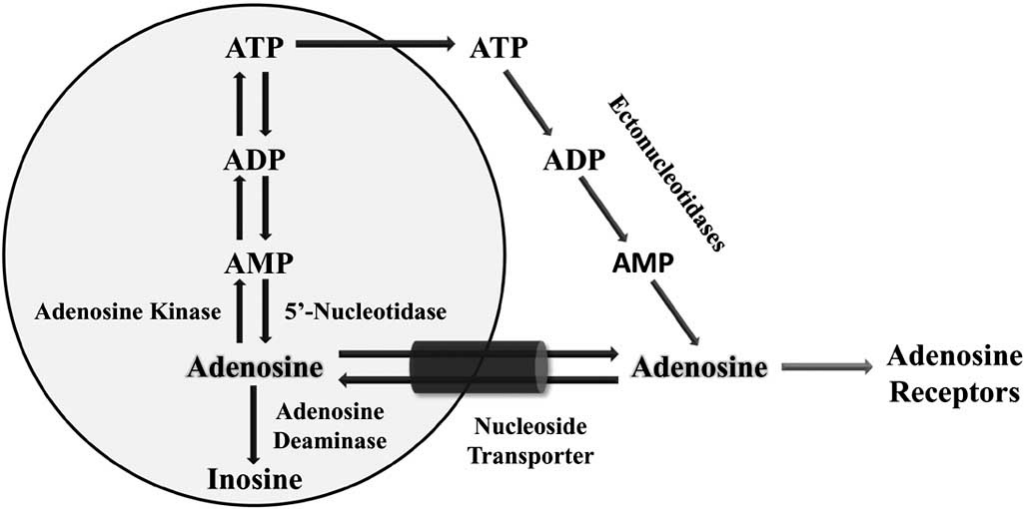
The principal source of adenosine in extracellular fluid spaces is equilibrative nucleoside transport, with the net direction of transport being dependent upon the concentration gradient of adenosine across the cell membrane. Another source of adenosine is the activity of ectonucleotidases that breakdown extracellular ATP to adenosine. Intracellular adenosine is formed from S-adenosyl homocysteine *via* SAH hydrolase (pathway not shown). Enzymes contributing to the hydrolytic cascade that converts ATP to adenosine include NTPDases and ecto-5’- nucleotidase. Adenosine produced by extracellular ATP hydrolysis or transported from the intracellular compartment acts on adenosine receptors on target cells in a paracrine or autocrine fashion. Clearance of adenosine from the extracellular space is provided by nucleoside transporters. Intracellular adenosine is hydrolysed by adenosine deaminase to inosine, or phosphorylated to AMP by adenosine kinase (ADK), which appears to be a major regulator of ambient adenosine levels.

The sources of adenosine in cochlear tissues include nucleoside transport from the intracellular compartment, extracellular nucleotide hydrolysis and release from damaged cells [53,113]. Extracellular adenosine levels are dynamically regulated by the release and reuptake of adenosine across the cell membrane *via* nucleoside transporters which have been identified in cochlear tissues [53]. Another potential source of adenosine is the activity of ectonucleotidases that breakdown extracellular ATP to adenosine [108-110]. Noise stress triggers the hydrolysis of ATP and generation of AMP, which is further dephosphorylated into adenosine by ecto-5’-nucleotidase [111]. Released adenosine is hydrolysed or removed from the extracellular space by nucleoside transporters [53]. Intracellularly, adenosine is hydrolysed by adenosine deaminase to inosine, whilst adenosine kinase (ADK), catalyses intracellular phosphorylation of adenosine to AMP. Based on its low *K*_M_ for adenosine, ADK is likely a major regulator of ambient adenosine levels in the extracellular space (Fig. **2**).

The extracellular concentrations of adenosine in cells and tissue fluids are quite low under physiological conditions (in the nanomolar range), whereas in different forms of cellular distress adenosine levels can reach as high as 100 μM [31,41]. In comparison, levels of intracellular ATP are 5-10 mM under physiological conditions. Because the intracellular concentration of ATP is so much higher than that of adenosine, slight changes in ATP concentration will result in substantial changes in adenosine levels [18,30]. Damage to cell membranes during trauma causes massive release of ATP into extracellular spaces and adenosine generation after ATP dephosphorylation by membrane-bound NTPDases and ecto-5’-nucleotidase [111,112]. Both purines may have an otoprotective role under different stress conditions [59].

## ADENOSINE TRANSPORT IN THE COCHLEA

Nucleoside transport appears to be essential for the regulation of adenosine concentrations in the cochlear fluids [53] where it is available to influence cell function through its action on adenosine receptors. In most tissues, principal nucleoside transport is mediated by equilibrative bidirectional transporters, with the net direction of transport being dependent upon the concentration gradient of adenosine across the cell membrane [4]. Because these transporters equilibrate the levels of intracellular and extracellular adenosine, changes in the level on one side can alter the level on the opposite side of the membrane. The equilibrative nucleoside transporter (ENT) family has four members (ENT1-4). ENT1 and ENT2 show differential sensitivity to nitrobenzylthioinosine (NBMPR). NBMPR-sensitive ENT1 and NBMPR-insensitive ENT2 have broad substrate specificity and tissue distribution [5,55], whilst ENT3 and ENT4 have unknown function. There is also a group of concentrative nucleoside transporters (CNTs) that transport adenosine against the concentration gradient [4]. In contrast to facilitated carrier proteins, the CNTs are Na^+^-dependent uphill transporters. Na^+^-dependent CNTs require energy from Na^+^-K^+^-ATPase to transport substrates into cells against their concentration gradient. CNT1 prefers pyrimidine nucleosides, CNT2 purine nucleosides and CNT3 transports both pyrimidine and purine nucleosides [37]. At least four types of nucleoside transporter are expressed in the cochlea [53] including two equilibrative transporters (ENT1 and ENT2) and two concentrative transporters (CNT1 and CNT2) encoded by the gene families SLC28 and SLC29, respectively. Adenosine uptake studies show that adenosine transport in the cochlea represents the net activity of Na^+^-dependent CNTs and Na^+^-independent ENTs [53]. The equilibrative carriers acting *via* facilitative diffusion enable an intracellular influx of adenosine based on its concentration gradient. In contrast, concentrative (uphill) nucleoside transporters counter gradient-dependent adenosine uptake by releasing adenosine from the tissue, and this is likely a *modus operandi *during cellular distress. In addition to regulating the actions of adenosine at P1 receptors, adenosine reuptake enables purine salvage in the cochlea and serves the maintenance of purinergic homeostasis.

## ADENOSINE RECEPTORS, COCHLEAR BLOOD FLOW AND SOUND-EVOKED POTENTIALS

Perilymphatic perfusion of adenosine (1 μM - 10 mM) in the guinea-pig cochlea induces a dose dependent increase in cochlear blood flow [72]. The blood flow response to adenosine is abolished in the presence of theophylline, a broadly specific adenosine receptor antagonist [72]. This effect of adenosine on cochlear blood flow is likely mediated by A_2A_ receptors based on their distribution in cochlear blood vessels [113]. The control of cochlear blood flow is important as compromised blood flow during chronic noise exposure and aging may lead to reduced auditory sensitivity [70,94].

Application of adenosine to isolated guinea-pig inner hair cells produces a small rise in intracellular Ca^2+^ [23]. However, auditory potentials in the intact animal are seemingly not affected by adenosine receptor agonists. The non-hydrolysable adenosine analogue *R*-phenylisopropyladenosine (*R*PIA) applied on to the round window membrane does not affect the endocochlear potential which drives the sensory transduction and has no influence on sound evoked auditory thresholds (e.g. auditory brainstem responses and compound action potential) [27]. Likewise, endolymphatic microinjections of adenosine (100 μM – 1 mM) in the guinea-pig cochlea do not change endocochlear potential and cochlear microphonic [73]. These data suggest that adenosine has limited effects on normal cochlear function, but further studies are required to identify cochlear responses to more selective adenosine receptor agonists than are currently available.

Even though adenosine receptor stimulation does not affect auditory potentials, a role of adenosine signalling in cochlear protection from oxidative stress has received a considerable attention in recent years. The next section will highlight the attempts to prevent cochlear injury by using selective agonists of adenosine receptors.

## NOISE STRESS AND OTOPROTECTION BY ADENOSINE

Adenosine receptor agonists have been successfully used in the treatment of ischemic brain and cardiac injury and are proving to have extraordinary cytoprotective functions. In the central nervous system, purinergic mechanisms are involved in various pathological conditions, including stroke, epilepsy, migraine, neurodegenerative and neuropsychiatric diseases [12,48]. The extensive background of neuroprotective action suggests that adenosine may also confer protection from noise-induced hearing loss, which has underlying mechanisms similar to some CNS pathologies.

There is increasing evidence that oxidative stress and the production of reactive oxygen species (ROS) are key elements in the pathogenesis of many forms of cochlear injury, for example from noise exposure, cytotoxic drugs and with aging. Indeed oxidative stress, along with neurotoxicity of glutamate, is being viewed almost as a unifying mechanism underlying most cochlear damage and hearing loss. Thus compounds that target mechanisms underlying oxidative stress offer considerable potential as therapies for hearing loss.

Noise exposure is a major cause of injury to the cochlea and hearing loss. It can be caused by an acute exposure to loud sound as well as by repeated exposure to moderate sounds over an extended period of time. Explosions and impulsive sounds can result in immediate hearing loss, whilst continuous exposure to loud noise, as experienced in the workplace or with recreational activities, likely damages the cochlea by slower metabolic mechanisms. Chronic exposure to high levels of noise can cause damage to the sensitive hair cells of the inner ear as well as the auditory nerve. Exposure to impulse or continuous noise may cause permanent or temporary hearing loss. The term ‘temporary threshold shift’ (TTS) has been used to indicate a transient impairment of auditory function due to noise trauma, and it usually disappears within few days after exposure to loud noise. Permanent threshold shift (PTS) occurs when post-exposure hearing thresholds have been stabilized at elevated levels.

Noise exposure drives mitochondrial activity and free radical production, reduces cochlear blood flow, causes excitotoxic swelling of afferent nerve terminals, and induces both necrotic and apoptotic cell death in the organ of Corti [42]. The formation of reactive oxygen species (ROS) results from increased mitochondrial activity [77]. ROS are molecules, ions and free radicals such as the superoxide ion, hydrogen peroxide, peroxynitrite, and hydroxyl radical. Under normal conditions, ROS produced by the mitochondria are easily metabolized or scavenged by endogenous antioxidant mechanisms. However, in the presence of excess ROS, the intrinsic cochlear defenses may be insufficient to control ROS production. ROS are formed as by-products of several metabolic pathways and these molecules can cause damage by reacting with DNA, proteins, membrane lipids, cytosolic molecules and cell surface receptors. The common ROS-initiating events are alterations of intracellular Ca^2+^ homeostasis and glutamate excitotoxicity [56]. NADPH oxidase may have a pivotal role in ROS formation in the cochlea induced by noise exposure [81]. ROS-induced changes in the cochlea include lipid peroxidation with the formation of malondialdehyde and 4-hydroxynonenal (4-HNE), inactivation of antioxidant enzymes and depletion of glutathione [83], leading to abnormalities in cochlear function [15]. Lipid peroxidation provides a link between the initial biochemical events in noise trauma and downstream intracellular pathways leading to apoptotic cell death. The generation of lipid peroxides appears to be highly correlated with pathophysiological findings in the noise-exposed cochlea [76].

Cells protect themselves against oxidative stress *via* an antioxidant defense system that maintains the appropriate redox state of cellular proteins. Antioxidant defenses can be augmented by a variety of molecules, including ROS scavengers (superoxide dismutase, catalase and glutathione peroxidase), trophic factors, protease inhibitors, lipid peroxidation inhibitors, heat shock proteins, and inhibitors of apoptosis [3,56,75,76].

Released adenosine may account for anti-oxidative defense and regeneration in a range of tissues [31,48,62]. Adenosine release in the cochlear fluid spaces is stimulated by oxidative stress [8] and by the activation of excitatory neurotransmission *via* NMDA receptors [92] densely distributed at the IHC afferent nerve endings. Ectonucleotidases NTPDase1, NTPDase2 and NTPDase3 are up-regulated during noise exposure, generating more adenosine from ATP hydrolysis by coupling to ecto-5’-nucleotidase [111,112]. In addition, oxidative stress upregulates the A_1_ adenosine receptor expression in the chinchilla cochlea *via* activation of NF-κB[51,81], whilst nitric oxide serves as an endogenous regulator of A_1_ receptors [50]. Round window application of the A_1_ receptor agonist *R*-PIA in chinchilla cochlea enhances activity of the two principal antioxidant enzymes in the cochlea, superoxide dismutase and glutathione peroxidase and reduces the levels of malondialdehyde, a marker of lipid peroxidation [27]. The pretreatment of the cochlea with *R*-PIA attenuates noise-induced hearing loss in animals exposed to a 4 kHz octave band noise (105 dB SPL for 4h) [47]. The reduction of permanent threshold shift is associated with reduced outer hair cell loss in R-PIA treated ears, suggesting that A_1_ receptors facilitate the recovery process of the outer hair cells after noise exposure. In addition, the combination of *R*-PIA with the antioxidant glutathione monoethylester provides otoprotection against both impulse and continuous noise in the chinchilla cochlea [43]. These findings support the otoprotective role of adenosine A_1_ receptors which have the capacity to increase the production of antioxidants and counter the toxic effects of ROS and glutamate [43,47,81].

## CISPLATIN OTOTOXICITY AND ADENOSINE 

Pharmacological targeting of A_1_ receptors is a novel approach to the prevention and treatment of ototoxicity. Two major classes of therapeutic agents can induce acquired hearing loss: aminoglycoside antibiotics and cisplatin-type chemotherapy agents [90]. These drugs target the outer hair cells particularly in the basal region of the cochlea to cause high frequency sensorineural hearing loss. Generation of ROS appears to be the principal mechanism of ototoxicity [91], triggering downstream cell death signalling pathways. Cisplatin is widely used to treat malignancies ranging from testicular, ovarian and bladder cancers to lung, head and neck malignancies. Audiometric measurements indicate that most patients develop at least some degree of hearing loss [91]. Elderly patients and children under 5 years of age are particularly susceptible to cisplatin ototoxicity [61]. Cisplatin affects sensory hair cells involved in sound transduction, spiral ganglion cells involved in auditory neurotransmission, and the lateral wall tissues (spiral ligament and stria vascularis) involved in K^+^ secretion and cycling. Cisplatin induces ROS generation in cochlear tissues and upregulation of the NOX3 isoform of NADPH oxidase [6]. This leads to increased production of superoxide ions, followed by formation of more toxic hydroxyl radical that interacts with polyunsaturated fatty acids in the cell membrane to form toxic aldehyde 4-hydroxynonenal (4-HNE). In addition, superoxide can react with nitric oxide to generate peroxynitrite radical which reacts with cell membrane proteins to form nitrotyrosine (NT). NT and 4-HNE are frequently used markers of free radical damage in the cochlea [52,121].

Even though the cochlea has endogenous mechanisms to deal with oxidative stress, these can be overwhelmed by excessive ROS production induced by cisplatin. In experimental conditions, cisplatin ototoxicity can be prevented by ROS scavengers (e.g. ebselen and N-acetylcysteine), neurotrophine-3, inhibitors of caspase 3 and 9, p53 inhibitor [89,91]. However, there is a concern that these drugs can interfere with the anti-tumor activity of cisplatin [7], hence alternative approaches have been sought. It has been shown that systemic sodium salicylate [60], or combination of ebselen and allopurinol [64,65] can protect against cisplatin ototoxicity without altering its antineoplastic activity.

Cisplatin treatment induces a fivefold increase in A_1_ receptor expression in the cochlea under experimental conditions [28], and the local application of adenosine A_1_ receptor agonists *R*-PIA or 2-chloro-N6-cyclopentyladenosine (CCPA) onto the round window membrane of the cochlea reduces cisplatin-induced auditory threshold shifts [118]. Protective effects can be reversed by pre-administration of the A_1_ receptor antagonist 1,3-dipropyl-8-cyclopentylxanthine (DPCPX). Despite encouraging initial data about the protective effects of adenosine receptor agonists on cisplatin ototoxicicty, further studies are required to demonstrate that systemic application of selective A_1_ receptor agonists does not interfere with anti-cancer effects of cisplatin.

## AGING AND OTOPROTECTION BY ADENOSINE

The term presbyacusis encompasses all conditions that lead to hearing loss in elderly people. The disorder is characterised by reduced hearing sensitivity and speech understanding in noisy environments, slowed central processing of acoustic information, and impaired localisation of sound sources [35]. The mechanisms of age-related hearing loss are poorly understood, but there is evidence that both genetic and environmental factors play a role. Presbyacusis can be regarded as a mixture of acquired auditory stresses, trauma, and otological diseases superimposed upon a genetically controlled aging process [35]. Some people and animal strains show greater resistance to hearing loss, which may reflect differences in their response to stressors such as noise exposure.

A number of mechanisms have been proposed to explain age-related cochlear injury. A dominant theory is that the damage arises from production of ROS [93], possibly as a consequence of impaired blood flow [20] or environmental factors such as excessive noise exposure [96]. This leads to membrane damage and damage to mitochondrial DNA [26,80,95,105]. Increased prevalence of apoptosis in cochlear hair cells in aged mice [106] and Mongolian gerbil [2] suggests that hair cell death is associated with activation of apoptotic pathways.

In the central nervous system, pathologies that respond well to adenosine drugs (e.g. Parkinson’s and Alzhemier’s disease, ischemic conditions and sleep disorders) are prevalent in elderly. In aged animals, expression levels and density of A_1_ adenosine receptors decrease in some brain regions, whilst the density of A_2A_ adenosine receptor increases (17,18,63,84]. The metabolism of adenosine is also altered in the aging brain [18], implicating dysfunctional adenosine homeostasis. Similarly, adenosine signalling and adenosine metabolism are altered in the heart of older animals leading to reduced capacity for cardioprotection [119]. Assuming that expression levels and density of adenosine receptors in the aging cochlea may undergo comparable changes, restoring the youthful balance of adenosine signalling by molecular and pharmacological means has a potential to diminish the progress of age-related hearing loss.

## ADENOSINE AS AN ANTI-INFLAMMATORY AGENT IN THE COCHLEA 

Sensorineural hearing loss and balance disorders are well known complications of meningitis [69] and otitis media, the prominent ear pathology of young children [13,78]. Middle ear infections can induce the inflammation of inner ear tissues by spread of toxins and bacteria through the round window membrane [19] whilst the meningeal infection spreads through the cochlear aqueduct, modiolus or hematogenically [19,69]. Labyrinthitis, or inflammation of the inner ear, can be caused by bacteria or viruses and is characterised by infiltration of inflammatory cells and damage to the inner ear tissues [78]. Environmental insults such as noise trauma can also cause an inflammatory response by the production of pro-inflammatory cytokines [32], which may exacerbate or induce further cochlear injury through a number of mechanisms, including metabolic pathways that involve oxidative stress [42]. A cochlear inflammatory response initiated in response to acoustic trauma involves the recruitment of circulating leukocytes to the inner ear [40,44,102]. While macrophages and other leukocytes may contribute to cochlear repair, the production of pro-inflammatory cytokines is also important in cochlear injury [32]. Similar mechanisms likely also contribute to the pathophysiology of age-related hearing loss [35,85].

During metabolic stress in other tissues, released adenosine limits excessive infiltration of neutrophils and mononuclear phagocytes and the release of pro-inflammatory cytokines [25]. Even though cells involved in immune and inflammatory responses (T cells, macrophages, dendritic cells) express all four adenosine receptor subtypes (A_1_, A_2A_, A_2B_, A_3_) [9], the A_2A_ receptor has been singled out as the principal modulator of inflammatory responses [57,68,98]. A_1_ receptors on macrophages also play a protective role in inflammation [99,104], in addition to their role in tissue protection and regeneration [30,31,62].

In some instances, inflammatory responses become deleterious [66] and require anti-inflammatory treatment. Current treatments of cochlear inflammation are based on steroids, which have positive short-term effects. However, long term steroid treatment is known to have a negative effect and can be pro-inflammatory. Adenosine-based treatment strategies based on the anti-inflammatory potential of A_2A_ adenosine receptors could represent powerful tool in limiting cochlear inflammation and consequent hearing loss.

## PUTATIVE MECHANISMS OF OTOPROTECTION BY ADENOSINE

A number of studies have demonstrated that cochlear injury caused by noise exposure and ototoxic drugs can be prevented or reduced by drugs targeting A_1_ adenosine receptors. Adenosine mechanisms accounting for tissue protection are numerous. They improve cochlear blood flow and oxygen supply, increase the production of antioxidants, and counter the toxic effects of ROS [31,62]. Adenosine can limit inflammatory responses [25,30] and provide vascular growth in areas with reduced oxygen tension [1]. Angiogenesis may be important in cochlear repair after injury and angiogenic factors such as adenosine may be useful in therapy of chronic inner ear pathologies.

Other mechanisms underlying adenosine receptor-mediated cytoprotection includes inhibition of glutamate release *via* presynaptic A_1_ receptors [30]. Activation of the presynaptic A_1_ receptors reduces Ca^2+^ influx through the preferential inhibition of N-type and Q-type channels [120,122]. The postsynaptic effects of A_1_ receptors include direct hyperpolarisation of neurones *via* G protein-activated inwardly rectifying K^+^ channels Kir 3.2 and 3.4 [100]. These mechanisms may lead to reduced cochlear sensitivity in the time of stress. In contrast, selective stimulation of adenosine A_2A_ receptors increases excitatory amino acid release [17] and may contribute to cochlear injury.

To date, the role of adenosine receptors in mediating the cochlear response to stress and their therapeutic potential are still vague. Stimulation of A_1_ receptors can prevent acute onset inner ear pathologies, but prolonged activation can cause receptor desensitisation and down-regulation which can hamper their neuroprotective potential in chronic conditions [18]. Translation research using A_1_ receptor agonists to provide otoprotection and neuroprotection has been confounded by their profound cardiovascular effects (bradycardia, hypotensia and hypothermia) and poor blood-brain barrier permeability upon systemic administration [11,97]. On the bright side, selective A_1_ adenosine receptor agonists have been developed with reduced peripheral side effects and increased ability to cross the blood-brain barrier [49]. Some of these adenosine receptor prodrugs hold promise for systemic interventions in the inner ear pathologies.

A_2A_ and A_3_ receptors have a different role in cochlear response to oxidative stress. The stimulation of A_2A_ receptors aggravates drug-induced cochlear injury [1^18^], suggesting an inhibition of A_2A_ adenosine receptors as attractive strategy for therapeutic management of chronic inner ear conditions. As for the A_3_ receptors agonists, they have a potential to promote tissue survival at lower concentrations [27], but may induce apoptosis at higher concentrations [33], making them less viable therapeutic options.

## CONCLUSION AND FUTURE STRATEGIES

Currently there are very few interventions for sensorineural hearing loss, apart from hearing aids which do not restore normal hearing. Hearing conservation programmes to reduce hearing loss from noise exposure have been only marginally successful. Thus the search for new treatments and interventions is essential to reduce the incidence and impact of the hearing disability. Extracellular adenosine, acting *via* P1 receptors, provides a primary signal transduction pathway that could be used to affect the progression of cochlear stress-induced pathology. The distribution of adenosine receptors in sensory and neural tissues of the cochlea, A_1_ receptor up-regulation during noise exposure, and reduced noise-induced cochlear injury after pretreatment with A_1_ receptor agonists suggest that adenosine receptors are well positioned to provide cochlear protection from loud sound and promote recovery from noise exposure and other forms of oxidative stress. Multiple cytoprotective actions of adenosine in other tissues suggest that it has the potential to counteract the effect of oxidative stress which is the basis of many forms of cochlear injury that cause hearing loss.****

In addition to A_1_ receptor associated pathways, potential adenosine based otoprotective strategies include:

- **Combined Inhibition of A_2A_ Receptors and Adenosine Kinase**

Coupling A_2A_ receptor and adenosine kinase inhibition, which increases endogenous adenosine levels, may be the most robust neuroprotective strategy based on adenosine signalling system [18]. The inhibition of adenosine kinase and A_2A_ receptors can be effective over a long period of time without apparent side effects [36], and may be particularly effective in elderly [18].

- **Inhibition of Adenosine Uptake by Nucleoside Transporters**

The selective inhibition of adenosine uptake may prove clinically relevant in cochlear protection, as it may serve to increase extracellular adenosine concentrations [53].

- **Increasing Adenosine Production from ATP**

The up-regulation of ectonucleotidase activity in cochlear fluid spaces serves not only to limit ATP signalling, but also to increase adenosine production and consequently cochlear resistance to oxidative stress [111].

- **Manipulating Adenosine Metabolism**

Adenosine kinase and adenosine deaminase are the principal enzymes involved in regulation of intracellular and extracellular adenosine levels and their inhibition may have a potential to ameliorate noise-induced cochlear injury and restore hearing.

## Figures and Tables

**Table 1.** Overview of Adenosine Receptor Tissue Distribution in the Rat Cochlea

	A_1_R	A_2A_R	A_3_R
Organ of Corti			
Inner hair cells	++	++	+
Outer hair cells			+
Deiters’ cells	++	++	++
Pillar cells			++
Hensen cells			+
Claudius cells			+
Epithelial cells			
Innner sulcus cells			++
Outer sulcus cells			++
Lateral wall			
Spiral ligament		++	+
Stria vascularis			
Spiral ganglion neurones	+	++	++
Blood vessels		++	

+ light immunostaining

++ strong immunostaining
